# Transhepatic Venous Angioplasty and Stenting: A Treatment Option in Bleeding from Gastric Varices Secondary to Pancreatic Carcinoma

**DOI:** 10.1155/1997/18272

**Published:** 1997

**Authors:** J. M. Ferguson, K. R. Palmer, O. J. Garden, D. N. Redhead

**Affiliations:** 1 Departments of Radiology and Surgery Western General Hospital Edinburgh Scotland; 2 Royal Infirmary of Edinburgh Western General Hospital Edinburgh Scotland; 3 the Gastro-intestinal Unit Western General Hospital Edinburgh Scotland

## Abstract

We present a case of recurrent variceal bleeding due to subtotal occlusion of the splenoportal junction by a pancreatic carcinoma. This was effectively treated by transhepatic venous angioplasty and stenting.


*HPB Surgery*, 1997, Vol.10, pp. 173–175
Reprints available directly from the publisher
Photocopying permitted by license only

© 1997 OPA (Overseas Publishers Association)
Amsterdam B.V. Published in The Netherlands
by Harwood Academic Publishers
Printed in India

### CASE REPORT
### Transhepatic Venous Angioplasty and Stenting: A Treatment Option in Bleeding from Gastric Varices Secondary to Pancreatic Carcinoma

J.M. FERGUSON, K.R. PALMER#, O.J. GARDEN* and D.N. REDHEAD

*Departments of Radiology and Surgery, #Royal Infirmary of Edinburgh and the Gastro-intestinal Unit, Western General Hospital, Edinburgh

*(Received 1 September 1994)*

We present a case of recurrent variceal bleeding due to subtotal occlusion of the splenoportal junction by a pancreatic carcinoma. This was effectively treated by transhepatic venous angioplasty and stenting.

KEY WORDS: Pancreatic carcinoma gastric varices transhepatic angioplasty stent

### CASE REPORT

A previously fit 71 year old male presented with a one month history of painless obstructive jaundice and malaise. Initial investigations confirmed obstructive jaundice with a bilirubin of 113 μmol/l (normal: 2–17 μmol/l). Haemoglobin was normal. Abdominal ultrasound demonstrated both intra-hepatic and extra-hepatic biliary dilatation and a hypoechoic lesion in the head of the pancreas measuring 2 × 3 cms. Computed tomography (CT) demonstrated early invasion of the superior mesenteric vein and guided fine needle aspiration cytology which confirmed the presence of a primary pancreatic adenocarcinoma. Jaundice was relieved by endoscopic biliary stent insertion using a 10 cm "Amsterdam" plastic stent.

6 months after initial presentation he was admitted with acute upper GI bleeding due to gastric varices demonstrated on endoscopy and treated by injection with ethanolamine. He experienced repeated episodes of bleeding over 4 weeks despite further endoscopic injections of the varices with histoacryl.

A mesenteric angiogram and indirect portogram demonstrated subtotal occlusion of the portal vein at the spleno-portal junction due to tumour involvement. (Fig. 1) The lesion was crossed using a transhepatic approach and following balloon angioplasty a 12 mm diam. Wallstent (Schneider (Europe) AG, Switzerland) was placed across the splenoportal junction from portal vein to splenic vein. (Fig. 2) The transhepatic track was embolised with coils. There were no immediate complications following the procedure and there was no recurrence of bleeding by the time of death 18 weeks later.

### DISCUSSION

Splenic vein stenosis or occlusion secondary to direct invasion by pancreatic carcinoma is well recognised although uncommon.[1] It also occurs in association with acute and chronic pancreatitis varying in incidence from 9% to 54% but it is frequently overlooked.[2,3] As a consequence of the splenic vein occlusion venous flow occurs via the short gastric veins to the stomach and through submucosal veins within the stomach wall to the coronary vein and hence to the portal vein. Dilatation of this venous collateral system produces isolated gastric varices which suggest splenic

*Correspondence to*: Dr. D.N. Redhead, Consultant Radiologist, Radiology Department, Royal Infirmary of Edinburgh, Lauriston Place, Edinburgh EH3 9YW.

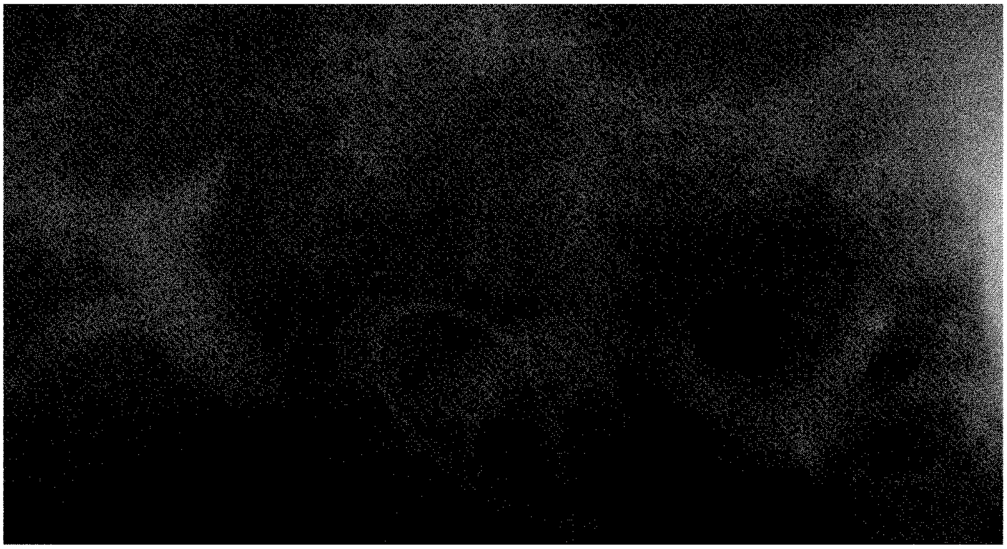

**Figure 1** Splenic arteriogram during venous phase demonstrating subtotal occlusion of portal vein at spleno-portal junction.

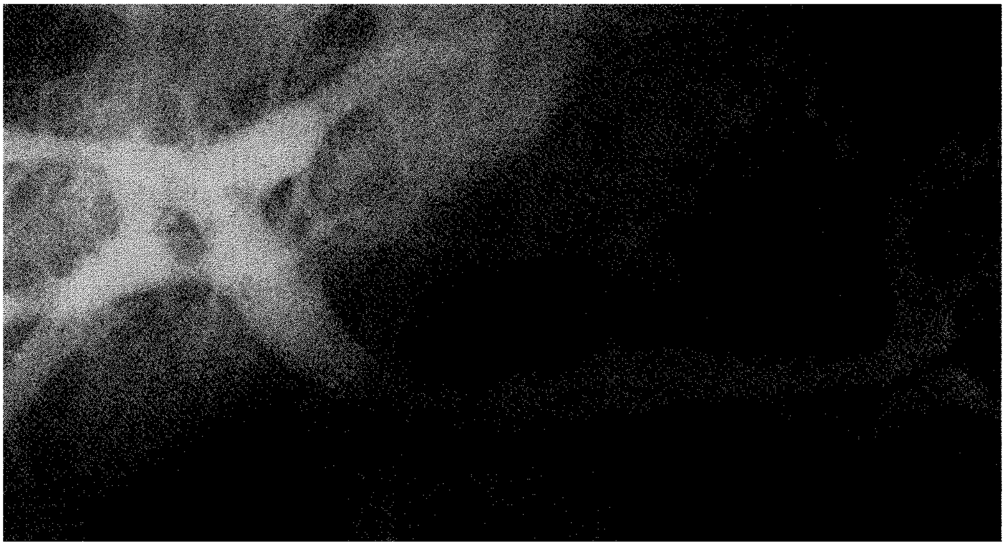

**Figure 2** Direct portogram following stent placement demonstrating resolution of portal vein stenosis and excellent flow in the hepatic portal vein branches.

vein occlusion. When the coronary vein drains into an occluded splenic vein or splenoportal junction venous flow occurs via the oesophageal veins to the azygous system leading to the development of both oesophageal and gastric varices.

It is important to recognise left sided portal hypertension as treatment is quite different from portal hypertension secondary to cirrhosis where pressure in the portal vein is raised. Indeed segmental portal hypertension in cirrhosis is uncommon, occurring in less than 5% of cases, and is always associated with signs of generalised portal hypertension.[4]

The diagnosis of gastric varices is problematic since the posterior wall of the gastric fundus is a difficult area to view endoscopically especially in the presence of acute bleeding. Varices may be mistaken for prominent gastric folds if the diagnosis is not suspected. However, endoscopy remains the mainstay of diagnosis and allows therapeutic sclerotherapy to be undertaken, as in the case described. Gastric varices can be demonstrated with a double contrast barium meal in up to 89% of cases but is inappropriate in the acute setting as residual barium will interfere with any subsequent angiography.[5] Arterial portography will confirm splenic vein occlusion and demonstrate the varices.[6] Definitive treatment for isolated gastric varices due to splenic vein occlusion is usually by splenectomy which is curative in 90% of cases. This can be combined with gastrotomy and oversewing of the varices.[4,6] Splenectomy is inappropriate as treatment of gastric variceal bleeding from portal hypertension due to cirrhosis when the splenic vein is patent. In such cases surgical treatment may consist of a devascularisation procedure which entails undersewing of the gastric varices with ligation of either the left gastric pedicle, the perioesophageal veins or the short gastric vessels or a combination of these. Surgical portosystemic shunting can be used but transjugular intrahepatic portosystemic stent shunt (TIPSS) is now a common and successful treatment in portal hypertension due to cirrhosis.[7] Splenectomy was considered inappropriate in our patient given his poor long term outlook with a locally advanced pancreatic carcinoma and the potential morbidity and mortality of surgery. Endovascular metallic stents are now well established in the treatment of venous occlusion due to malignant disease in the superior vena cava.[8] Successful treatment by angioplasty of portal vein stenosis following orthotopic liver transplant has been described[9] as well as in obstruction due to pancreatitis. [10] More recently a case of portal vein thrombosis post liver transplant was successfully treated by thrombolysis and dilatation of the underlying portal vein stenosis with two overlapping Wallstents.[11] Cekirge at al described the use of a stent to treat splenic vein stenosis discovered after a TIPSS was created.[12]

Transhepatic venous angioplasty and stenting appeared to be the treatment of choice in the present case. Although no complications occurred as a consequence of the procedure we recommend aggressive correction of any coagulation abnormalities and embolisation of the hepatic track to limit the risk of bleeding. The present case demonstrates the value of stenting when established medical management of gastric variceal haemorrhage has been unsuccessful and surgery is considered inappropriate.

### REFERENCES

1. Sutton, J.P., Yarborough, D.Y. and Richards, J.T. (1970) Isolated splenic vein occlusion. *Archives of Surgery*, **100**, 623–626.
2. Leger, L., Lenriot, J. and Lemaigre, G. (1968) L'hypertension et la stase portales segmentaires dans les pancreatites chronique. *Journal de Chirurgie* (Paris), **95**, 599–608.
3. L'Hermine, C., Lemaitre, G., Maillard, J.P. and Toison, F.L. (1971) Hypertension portale segmentaire des pancreatites. Aspect angiographiques. *Lille Medical.*, **16**, 928–932.
4. Greig, J.D., Garden, O.J., Anderson, J.R. and Carter, D.C. (1990) Management of gastric variceal haemorrhage. *British Journal of Surgery.*, **77**, 297–299.
5. Muhletaler, C., Gerlock, A.J. Jr., Goncharenko, V., Avant, G.R. and Flexner, J.M. (1979) Gastric varices secondary to splenic vein occlusion: Radiographic diagnosis and clinical significance. *Radiology.*, **132**, 593–598.
6. Little, A.G. and Moossa, A.R. (1981) Gastrointestinal haemorrhage from left-sided portal hypertension: an unappreciated complication of pancreatitis. *American Journal of Surgery.*, **141**, 153–158.
7. Chalmers, N., Redhead, D.N., Simpson, K.J. and Hayes, P.C. (1992) Transjugular intrahepatic portosystemic stent shunt (TIPSS): early clinical experience. *Clinical Radiology*, **46**, 166–169.
8. Charnsangavej, C., Carrasco, C.H. and Wallace, S. (1986) Stenosis of the vena cava: Preliminary assessment of treatment with expandable metallic stents. *Radiology*, **161**, 295–298.
9. Olliff, S.P., Pain, J.A., Karani, J.B., Mowat, A.P. and Williams, R. (1991) Percutaneous transhepatic diltatation of portal vein stenosis following orthotopic liver transplantation. *Journal of Interventional Radiology*, **6**, 29–31.
10. Uflacker, R., Alves, M.A., Cantisani, G.G., Souza, H.P., Wagner, J. and Moraes, L.F. (1985) Treatment of portal vein obstruction by transhepatic angioplasty. *Gastroenterology*, **88**, 176–180.
11. Haskal, Z.J. and Naji, A. (1993) Treatment of portal vein thrombosis after liver transplantation with percutaneous thrombolysis and stent placement. *Journal of Vascular and Interventional Radiology*, **4**, 789–792.
12. Cekirge, S.H., Weiss, J.P., Foster, R.G. and McLean, G.K. (1993) Stent placement in a splenic vein stenosis after TIPS creation. *Radiology*, **189**, 197–198.

